# Drug-Induced Hyponatremia as a Cause of Emergency Department Attendance

**DOI:** 10.3390/pharmacy14020049

**Published:** 2026-03-17

**Authors:** Joel Gené Grasa, Natalia Sanz López, Marta Docio Alfaya, Verónica Mate García, Alicia Serrano García-Calvo, Adrián Plaza Díaz, Jesús Ruiz Ramos

**Affiliations:** 1 Pharmacy Department, Institut de Recerca Sant Pau (IR Sant Pau), Hospital de la Santa Creu i Sant Pau, 08041 Barcelona, Spain; jgene@santpau.cat (J.G.G.); aplaza@santpau.cat (A.P.D.); 2 Emergency Department, Institut de Recerca Sant Pau (IR Sant Pau), Hospital de la Santa Creu i Sant Pau, 08041 Barcelona, Spain; nsanzl@santpau.cat (N.S.L.); mdocio@santpau.cat (M.D.A.); vmate@santpau.cat (V.M.G.); aserranoga@santpau.cat (A.S.G.-C.)

**Keywords:** hyponatremia, emergency department, polypharmacy

## Abstract

Background: Hyponatremia is one of the most common electrolyte disturbances in emergency departments. This study aimed to describe the characteristics of patients presenting with drug-induced hyponatremia and analyse factors associated with 30-day revisits. Methods: Retrospective observational study including adult patients who attended the emergency department for drug-induced hyponatremia between 2020 and 2024. Results: 141 patients were analysed (mean age 80.5 years; 78% women). Thiazide diuretics were the most frequently implicated pharmacological class (50.3%). In univariable analyses, polypharmacy, dementia, and treatment changes at discharge were associated with a higher risk of revisit for hyponatremia. In the multivariable model, only polypharmacy remained significantly associated with 30-day revisits. Conclusions: Thiazide diuretics were the leading drug-related cause of hyponatremia in the emergency setting. Polypharmacy was identified as an independent factor associated with increased revisit risk, underscoring the need for systematic medication review and close clinical follow-up after hospital discharge.

## 1. Introduction

Hyponatremia, clinically characterized by serum sodium concentration falling below 135 mmol/L, ranks among the most prevalent electrolyte imbalances encountered in Emergency Departments (EDs) [1,2]. This condition poses significant health risks, including an elevated likelihood of falls, deterioration in functional status, and increased rates of hospital admissions [3]. The multifactorial nature of hyponatremia is underscored by its diverse etiologies, which encompass a range of endocrine and metabolic disorders, renal insufficiency, heart failure, various malignancies, and, critically, the adverse effects of medications frequently prescribed to manage comorbid health conditions [4].

Several studies have illuminated the correlation between specific pharmacological agents and the onset of hyponatremia [5]. Drug-induced hyponatremia is particularly relevant in the ED because it is often preventable and may recur if the culprit medication is not identified, discontinued, or adequately substituted. Thiazide diuretics, antidepressants, antiepileptics, and other agents can precipitate hyponatremia through different mechanisms, including renal sodium loss and impaired free-water excretion. Risk is amplified in older adults by reduced renal reserve, comorbidity burden, intercurrent acute illness, and polypharmacy with potential drug–drug interactions. Therefore, characterizing medication patterns and patient vulnerability is essential to guide safer prescribing and optimize discharge planning and follow-up strategies aimed at preventing early ED revisits. However, there remains a notable gap in the literature concerning the clinical and pharmacological determinants that contribute to revisits to the ED due to drug-induced hyponatremia. This gap is particularly concerning given that the demographic frequently presenting to EDs is predominantly the elderly, often on multiple medications and grappling with chronic health issues.

The primary objective of this study was to delineate the clinical, analytical, and pharmacological profiles of patients who arrive at the ED with hyponatremia. Furthermore, this research aimed to identify and analyze the risk factors that may predispose these individuals to return to the ED for management of this electrolyte disorder. Understanding these factors is pivotal in enhancing patient care and mitigating the complications associated with hyponatremia in vulnerable populations.

## 2. Materials and Methods

A retrospective observational study was conducted including patients (≥18 years) who presented to the ED of a tertiary care hospital with moderate or severe hyponatremia (<130 mmol/L) [6] as a primary or secondary diagnosis between 2020 and 2024. Patients were retrospectively identified using the hospital activity database, the electronic medical record, and the institutional registry of adverse drug events (ADEs). Only patients who were discharged alive from the index episode were eligible for the 30-day revisit analysis. Patients who died during the index admission, were transferred to another acute-care hospital, or had incomplete follow-up information were not considered at risk for revisits and were excluded (or censored) accordingly.

Suspected drug-induced hyponatremia was defined as hypotonic hyponatremia in which at least one medication with a plausible mechanism (e.g., thiazides, SSRIs, antiepileptics) was identified as a likely contributor by the treating team and confirmed by structured chart review. Attribution required: (i) a compatible temporal relationship between drug exposure and hyponatremia; (ii) absence of a more plausible alternative explanation after standard ED evaluation; and (iii) documented sodium normalization or clear upward trend after drug withdrawal when follow-up data were available. Serum sodium was interpreted in the context of glycemia. When hyperglycemia was present, sodium was corrected for glucose using a standard correction factor to reduce the risk of misclassification. Cases were reviewed by an ED physician and a clinical pharmacist; disagreements were resolved by consensus.

The primary endpoint was an ED revisit for hyponatremia within 30 days after index discharge. Patients were followed from the date of discharge from the index ED/hospital episode until the first hyponatremia-related ED revisit or censoring or after 30 days. Data collection was carried out exclusively by the study investigators through direct manual review of medical records and hospital databases. Patients were identified by medical record number only, and no personally identifiable information was included in the study database. Data were stored in a secure Microsoft Excel database on the hospital’s institutional server, with access restricted to authorized investigators.

The following variables were recorded: age, sex, reason for ED presentation, patient origin, number of medications at admission, presence of dementia and degree of dependence, comorbidities, drug identified as the trigger for hyponatremia, serum sodium level at admission, plasma osmolality, urinary sodium concentration, urinary osmolality, discharge disposition, and whether treatment was modified at discharge.

At our institution, discharge documentation is protocolized using a standardized report template that includes: (1) primary and secondary discharge diagnoses; (2) structured medication reconciliation specifying medications to stop, start, and continue unchanged; and (3) standardized patient recommendations. For this study, these predefined discharge fields were reviewed to extract the recorded diagnoses, medication changes, and recommendations provided to patients. Functional dependence was obtained from the standardized nursing assessment recorded in the electronic medical record and categorized according to the documented level.

Hyponatremia severity was categorized according to guideline thresholds: moderate hyponatremia (125–129 mmol/L) and profound hyponatremia (<125 mmol/L) [7]. Serum sodium was interpreted in the context of glycemia. When hyperglycemia was present, sodium was corrected for glucose using a standard correction factor to reduce the risk of misclassification. Plasma osmolality was used to confirm true hypotonic hyponatremia (<275 mmol/L) and exclude pseudohyponatremia secondary to hyperglycemia or hyperlipidemia (>275 mmol/L). Urinary osmolality was used to guide etiological assessment: values < 100 mOsm/kg indicate appropriate suppression of antidiuretic hormone (ADH), whereas values > 100 mOsm/kg suggest inappropriate ADH secretion or drug-related ADH effect [8]. In the context of recent thiazide exposure, urine osmolality > 100 mOsm/kg together with urine sodium > 30 mmol/L supports impaired urinary dilution with renal sodium loss, consistent with thiazide-associated hyponatremia [9]. Volemic status at presentation (hypovolemic/euvolemic/hypervolemic) was classified using predefined criteria (physical examination findings, fluid balance, and available laboratory profile). Diagnostic work-up (including imaging such as CT) was performed at the discretion of the treating clinician according to presenting symptoms and standard ED practice. We recorded and documented comorbidities including active oncologic or hematologic disease and reviewed available records for alternative etiologies of hyponatremia.

Statistical analysis was performed using Stata version 13.0 (StataCorp, College Station, TX, USA). Initially, univariable logistic regression analyses were conducted using ED revisit as the dependent variable and each recorded variable as an independent predictor. Covariates were selected a priori based on clinical plausibility and prior literature, with additional variables entering the multivariable model when meeting pre-specified univariable criteria (*p* < 0.10). This study was conducted in accordance with current ethical regulations and approved by the institutional ethics committee (reference number: IIBSP-COD-2022-40).

## 3. Results

A total of 141 patients were included [mean age: 80.5 (SD: 12.5) years; 110 were female (78.0%)]. The median number of medications at admission was 8.5 (range 5–14), reflecting a predominantly polymedicated population. The most frequent comorbidities were hypertension (n = 121, 85%), diabetes mellitus (n = 53, 37%), and moderate dependence (n = 74, 53%). Most patients were admitted from home (n = 122, 86.5%). The most common reasons for ED presentation were dizziness, falls or syncope (n = 32, 22.7%), confusion or delirium (n = 20, 14.2%), and dyspnea or respiratory symptoms (n = 15, 10.6%) (Table 1).

Among included patients, 76 (53.9%) had severe hyponatremia (<125 mmol/L) and 65 (46.1%) had moderate hyponatremia (125–129 mmol/L). Urinary sodium was measured in 82 patients (58.2%), with values > 30 mmol/L in 66 cases (80.5%). Plasma osmolality was determined in 85 patients (60.3%), and values > 275 mOsm/kg were observed in 17 cases (20%). Volemic status was available in 120 (84.5%) cases; among these, euvolemia/hypovolemia/hypervolemia accounted for 47, 63 and 10 patients respectively. Treatment with the causative drug was discontinued after hospital discharge in 46 patients (32.9%). Mean potassium value was 3.9 (SD:0.6) mmol/L, presenting 34 (24.1%) patients with hypokalemia and 3 (2.1%) patients with hyperkalemia.

The drugs associated with hyponatremia episodes are summarized in Table 2. Percentages were calculated using the total number of implicated drugs as the denominator, as some patients were exposed to more than one suspected drug. Hydrochlorothiazide was the most frequently implicated agent, accounting for 79 cases (50.3%), whereas thiazide-like diuretics (e.g., indapamide, chlorthalidone) accounted for a smaller proportion. Less frequently, cases were associated with other therapeutic groups, including antidepressants (24 cases, 15.2%), antiepileptic drugs (12 cases, 7.6%), antihypertensive agents (3 cases, 1.9%), antibacterials (2 cases, 1.3%), hormones (1 case, 0.6%), and opioid analgesics (1 case, 0.6%).

Among patients who revisited the ED with hyponatremia within 30 days (n = 16), the mean glucose-corrected serum sodium was 127.2 (SD: 3.7) and the mean potassium was 4.8 (SD: 0.6). Hyponatremia was moderate in 8 (50.0%) patients and severe in 3 (18.7%). Results of the univariable analysis of factors associated with ED revisits for hyponatremia are shown in Table 3. Variables such as the use of more than ten medications at admission (OR 3.36; 95% CI 1.13–9.99), dementia (OR 2.69; 95% CI 0.88–8.27), and treatment modification at discharge (OR 2.65; 95% CI 0.90–7.82) reached a *p* value < 0.1 and were therefore included in the multivariate analysis.

In the multivariable model (Table 4), the use of more than ten medications at admission was significantly associated with ED revisit (OR 3.22; 95% CI 1.05–9.91; *p* = 0.041). In contrast, neither treatment modification at discharge (OR 2.49; 95% CI 0.81–7.70; *p* = 0.113) nor the presence of dementia (OR 2.16; 95% CI 0.66–7.05; *p* = 0.200) showed a statistically significant association. No other variables reached statistical significance in the multivariate model.

## 4. Discussion

Our results suggest that thiazide diuretics are the main pharmacological group responsible for ED visits due to hyponatremia. This highlights the critical need for healthcare providers to closely monitor medication regimens in elderly patients to mitigate the risk of recurrent hyponatremia and its associated complications.

The patients included in our study were predominantly elderly (>80 years), highly polymedicated, and predominantly female, a profile consistent with that of patients commonly presenting to EDs due to ADEs [10,11]. This combination defines a frail population in whom the onset and recurrence of electrolyte disturbances such as hyponatremia may present with nonspecific clinical manifestations, including dizziness, falls, confusion, or asthenia, which frequently prompt ED consultation [12,13], as observed in our cohort. Although euvolemic presentations were common in our cohort, hypovolemia remained the predominant volemic pattern. This likely reflects the high prevalence of diuretic exposure, particularly thiazides and loop diuretics, which can promote renal sodium losses and clinically apparent volume depletion. Moreover, in an older, frail ED population, concurrent precipitants such as reduced oral intake, intercurrent infection, and gastrointestinal losses are frequent and may shift the overall presentation toward hypovolemia.

Advanced age and polypharmacy may help explain our main findings. Both in the univariable and multivariable analyses, the use of more than ten medications was statistically associated with a higher likelihood of ED revisit for hyponatremia, suggesting that polypharmacy may increase the risk of recurrent episodes. Several authors have linked polypharmacy to increased hospital readmissions and ED visits [14,15]. Possible explanations include the concurrent use of drugs capable of inducing hyponatremia or other electrolyte disturbances, a higher likelihood of drug–drug interactions and medication errors, and increased frailty with limited compensatory mechanisms [16]. Although our study lacked sufficient power to evaluate associations by specific drug classes, the findings highlight the need to prioritize medication review at discharge and implement deprescribing strategies when clinically appropriate.

From a practical perspective, our findings have direct implications for ED discharge planning and transitional care in patients with suspected drug-induced hyponatremia. First, the predominance of hydrochlorothiazide and the frequent coexistence of potassium abnormalities (hypokalemia in about one quarter of patients) support a “medication-first” approach: prompt identification of the drugs involved, consideration of safer alternatives (switching from thiazides to non-thiazide antihypertensives when clinically appropriate), and correction of hypokalemia, which may perpetuate hyponatremia via renal sodium and water handling. Second, although hyponatremia is often described as euvolemic in older adults, most patients in our cohort were classified as hypovolemic, a pattern that may reflect diuretic exposure and intercurrent illness but also underscores the difficulty of bedside volume assessment in routine ED practice. This reinforces the value of explicitly documenting volemic status and, when available, integrating urinary indices (urine sodium and osmolality) to support etiologic classification and guide management decisions. Third, the independent association between polypharmacy and 30-day revisits suggests that recurrence is less driven by a single comorbidity than by cumulative medication burden and vulnerability to repeated iatrogenic electrolyte disturbances. Embedding pharmacist-led medication reconciliation and post-discharge follow-up may further reduce early revisits by ensuring adherence to medication changes, detecting recurrent symptoms, and expediting laboratory monitoring [17]. Future prospective multicenter studies should test whether bundled interventions reduce ED revisits and incorporate standardized causality adjudication and competing-risk methods to account for death or transfer during follow-up.

Although dementia showed a non-significant trend toward higher revisit risk, this finding should be interpreted cautiously. Importantly, hyponatremia itself can produce neurocognitive symptoms: acute reductions in serum sodium may present with delirium, confusion, and somnolence, whereas chronic hyponatremia has been associated with subtle but clinically meaningful cognitive dysfunction and impaired attention [18]. Consequently, patients with mild chronic hyponatremia may be considered asymptomatic, while neurocognitive effects are inadvertently attributed to other causes. In older adults, hyponatremia is frequently euvolemic, and the overlap between pre-existing cognitive impairment and hyponatremia-related cognitive manifestations may contribute to misclassification and confounding [19,20]. Therefore, the observed association may reflect diagnostic overlap and differences in outpatient management (medication adherence, access to monitoring, caregiver support) rather than dementia. Future prospective studies should incorporate standardized cognitive assessment and longitudinal sodium trajectories to better distinguish baseline cognitive impairment from hyponatremia-related neurocognitive effects.

Overall, our study did not identify statistically significant associations between ED revisits and individual comorbidities such as heart failure or chronic kidney disease, which have been linked to hyponatremia in previous studies. This may be attributable to several factors, including the moderate sample size, heterogeneity in clinical presentation, and the likelihood that the cumulative burden of multiple factors, rather than isolated comorbidities, determines the risk of ED revisit. The predominance of nonspecific presenting symptoms in our cohort further supports the concept that hyponatremia in the ED setting often presents as a multisystem and atypical condition in elderly patients.

This study has several limitations. Its retrospective and single-center design limits the generalizability of the findings. Although the sample size is reasonable for an ED cohort, it may be insufficient to detect certain associations. Because this is an observational design without a control group and with potential unmeasured factors (e.g., hydration status, severity of intercurrent illness, outpatient follow-up intensity), residual confounding is likely. In addition, drug-causality classification may be subject to measurement bias, although adjudication was performed by experienced clinicians using structured chart review. Because this was a retrospective study, formal rechallenge was not feasible and causality cannot be established; accordingly, our findings should be interpreted as associations within a cohort of suspected drug-induced cases. Because advanced imaging and malignancy screening were not protocolized, we cannot exclude the possibility that a proportion of cases labelled as drug-related were influenced by occult SIADH-related conditions, including malignancy. Despite these limitations, the observed trends toward increased ED revisit risk associated with high medication burden and treatment modification at discharge have important practical implications. These include the recommendation to perform systematic medication review in patients presenting with hyponatremia, adopt cautious and shared therapeutic decisions at discharge, and implement close follow-up, particularly in polymedicated patients and those with cognitive impairment. The implementation of clinical pharmacy interventions and early follow-up strategies, such as telephone monitoring or prompt outpatient review, may help identify early recurrences and prevent readmissions [21,22]. Future studies, preferably prospective and with larger sample sizes, should evaluate the effectiveness of polypharmacy reduction strategies and post-discharge follow-up protocols in reducing ED revisits for hyponatremia.

## 5. Conclusions

In our cohort of patients presenting to the ED with hyponatremia, thiazide diuretics were the most frequently implicated pharmacological group. The use of more than ten medications at admission was significantly associated with a higher risk of ED revisit within 30 days. These findings reinforce the importance of polypharmacy as a potential risk factor for recurrent hyponatremia and highlight the need to establish strategies for medication review and optimization, as well as structured follow-up plans after an episode of hyponatremia.

## Figures and Tables

**Table 1:** Characteristics of the study population.

Characteristics	Patients (n) = 141
Age, mean (SD)	80.5 (12.5)
Sex, n (%)	
Female	110 (78.0%)
Male	31 (22.0%)
Number of medications at admission, median; (Range)	8.5 (1–21)
Comorbidities, n, (%)	
Hypertension	121 (85.8%)
Moderate dependency	74 (52.5%)
Diabetes mellitus	53 (37.6%)
Dementia	30 (21.3%)
Atrial fibrillation	29 (20.6%)
Congestive heart failure	23 (16.3%)
Ischemic heart disease	19 (13.5%)
Chronic kidney disease stage 3–5	27 (19.1%)
Active onco-hematologic disease	13 (9.2%)
Origin, n (%)	
Home	122 (86.5%)
Nursing home	18 (12.8%)
Another hospital	1 (0.71%)
Reason for admission, n (%)	
Dizziness/fall/syncope	32 (22.7%)
Other	24 (17.0%)
Confusion/delirium	20 (14.2%)
Dyspnea/respiratory symptoms	15 (10.6%)
Asthenia/fatigue	14 (9.9%)
Somnolence	14 (9.9%)
Nausea/vomiting	7 (5.0%)
Gait disturbance	5 (3.6%)
Abdominal pain	5 (3.6%)
Edema	5 (3.6%)

**Table 2:** Drugs implicated in the hyponatremia episode.

Therapeutic Group	Drug	n (%)
C03—Diuretics	Hydrochlorothiazide	79 (50.3%)
	Spironolactone	8 (5.1%)
	Furosemide	7 (4.5%)
	Indapamide	6 (3.8%)
	Chlorthalidone	2 (1.3%)
	Eplerenone	2 (1.3%)
	Torasemide	1 (0.6%)
C09—Antihypertensives	Enalapril	2 (1.3%)
	Losartan	1 (0.6%)
H01—Hormones	Desmopressin	1 (0.6%)
J01—Antibacterials	Cotrimoxazole	2 (1.3%)
N02—Analgesics	Tramadol	1 (0.6%)
	Paracetamol	1 (0.6%)
N03—Antiepileptics	Carbamazepine	7 (4.5%)
	Eslicarbazepine	2 (1.3%)
	Lamotrigine	1 (0.6%)
	Zonisamide	1 (0.6%)
	Gabapentin	1 (0.6%)
N05—Antipsychotics	Risperidone	1 (0.6%)
	Quetiapine	1 (0.6%)
N06—Antidepressants	Mirtazapine	6 (3.8%)
	Sertraline	5 (3.2%)
	Citalopram	4 (2.5%)
	Venlafaxine	3 (1.9%)
	Paroxetine	2 (1.3%)
	Fluoxetine	2 (1.3%)
	Desvenlafaxine	1 (0.6%)
	Trazodone	1 (0.6%)

**Table 3:** Results of the univariable analysis.

Characteristics	*p* -Value	OR	IC95%
Age > 80 years	0.689	0.80	0.27–2.35
More than 10 medications	0.030	3.36	1.13–9.99
Female sex	0.268	0.52	0.16–1.65
Dementia	0.083	2.69	0.88–8.27
Moderate dependency	0.274	1.88	0.61–5.80
Hypertension	0.920	1.08	0.23–5.21
Diabetes	0.895	1.05	0.52–2.11
Heart failure	0.683	1.33	0.34–5.12
Atrial fibrillation	0.204	2.13	0.66–6.79
Ischemic heart disease	0.427	0.43	0.05–3.46
CKD stage III–V	0.929	1.06	0.28–4.06
Active oncologic or hematologic disease	0.129	1.00	0.32–5.01
Treatment change at discharge	0.078	2.65	0.90–7.82
Serum sodium at admission < 120 mmol/L	0.689	0.73	0.15–3.45

**Table 4:** Results of the multivariable analysis.

Characteristics	*p* -Value	OR	IC95%
More than 10 medications	0.041	3.22	1.05–9.91
Dementia	0.200	2.16	0.66–7.05
Treatment change at discharge	0.113	2.49	0.81–7.70

## Data Availability

No new data were created or analyzed in this study.

## Abbreviations

The following abbreviations are used in this manuscript:

ADR	Adverse drug event
ED	Emergency department
